# Antiviral Activity of Vacuolar ATPase Blocker Diphyllin against SARS-CoV-2

**DOI:** 10.3390/microorganisms9030471

**Published:** 2021-02-25

**Authors:** Michal Stefanik, Petra Strakova, Jan Haviernik, Andrew D. Miller, Daniel Ruzek, Ludek Eyer

**Affiliations:** 1 Department of Virology, Veterinary Research Institute, CZ-62100 Brno, Czech Republic; stefanik@vri.cz (M.S.); strakova.petra@vri.cz (P.S.); haviernik@vri.cz (J.H.); miller@vri.cz (A.D.M.); ruzekd@paru.cas.cz (D.R.); 2 Department of Chemistry and Biochemistry, Mendel University in Brno, CZ-61300 Brno, Czech Republic; 3 KP Therapeutics (Europe) s.r.o., CZ-61200 Brno, Czech Republic; 4 Institute of Parasitology, Biology Centre of the Czech Academy of Sciences, CZ-37005 Ceske Budejovice, Czech Republic

**Keywords:** Severe Acute Respiratory Syndrome Coronavirus 2 (SARS-CoV-2), vacuolar ATPase blocker, diphyllin, cleistanthin B, helioxanthin, antiviral activity, cytotoxicity

## Abstract

Severe Acute Respiratory Syndrome Coronavirus 2 (SARS-CoV-2) is a causative agent of the pandemic coronavirus disease 2019 (COVID-19), which has resulted in over two million deaths worldwide to date. Diphyllin and diphyllinosides are known as natural blockers of cellular vacuolar ATPases, and so can act as inhibitors of the pH-dependent fusion of viral envelopes with host cell endosomal membranes. Such pH-dependent fusion is a critical early step during the SARS-CoV-2 replication cycle. Accordingly, the anti-SARS-CoV-2 profiles and cytotoxicities of diphyllin, diphyllinoside cleistanthin B, and two structurally related compounds, helioxanthin 8-1 and helioxanthin 5-4-2, are evaluated here using in vitro cell-based assay systems. Neither helioxanthin exhibits any obvious anti-SARS-CoV-2 effects in vitro. By contrast diphyllin and cleistanthin B do exhibit anti-SARS-CoV-2 effects in Vero cells, with respective 50% effective concentrations (EC_50_) values of 1.92 and 6.51 µM. Diphyllin displays anti-SARS-CoV-2 effect also in colorectal adenocarcinoma (CaCo-2) cells. Moreover, when diphyllin is added at various times post infection, a significant decrease in viral titer is observed in SARS-CoV-2-infected Vero cells, even at high viral multiplicities of infection. Importantly, neither diphyllin nor cleistanthin B are found cytotoxic to Vero cells in concentrations up to 100 µM. However, the cytotoxic effect of diphyllin is more pronounced in Vero E6 and CaCo-2 cells. Overall, our data demonstrate that diphyllin and diphyllin analogues might be perfected as anti-SARS-CoV-2 agents in future preclinical studies, most especially if nanomedicine approaches may be invoked to optimize functional drug delivery to virus infected cells.

## 1. Introduction

Severe Acute Respiratory Syndrome Coronavirus 2 (SARS-CoV-2), formerly known as 2019 novel coronavirus (2019-nCoV), is a causative agent of coronavirus disease 2019 (COVID-19). SARS-CoV-2 was firstly identified from the respiratory tract of pneumonia patients in Wuhan, China, in late December 2019, after which the virus spread to numerous other countries around the world over the next few months [1]. As a result, on March 11, 2020, the World Health Organization (WHO) announced COVID-19 to be a new pandemic disease [1]. As of 1 February 2021, there have been more than 101 million confirmed cases of COVID-19 globally, including over two million deaths (https://covid19.who.int/, accessed on 1 February 2021). SARS-CoV-2 is an airborne virus, which is typically transmitted from person to person via respiratory droplets, although transmission through contaminated surfaces is also highly likely. The clinical course of COVID-19 can be complex. In most cases, the infection is asymptomatic or relatively mild, accompanied by fever, cough, shortness of breath, and muscle ache [2]. In some cases, however, particularly in elderly patients and those with cardiac and respiratory disorders, SARS-CoV-2 infection may progress to serious and life-threatening acute respiratory distress syndrome (ARDS) or pneumonia [3,4] resulting in sepsis, multiple organ dysfunction, and death [2]. Around 3–29% patients may need admission to intensive care units [5]. Although, several types of vaccination strategies are currently available, new highly anti-COVID-19 drugs are needed urgently to combat the disease. Numerous anti-COVID-19 clinical trials are ongoing seeking to repurpose known drugs against SARS-CoV-2 infections, including trials with remdesivir, favipiravir, or hydroxychloroquin [1]. However, the primary unmet medical need is for the development of genuine, small molecule anti- SARS-CoV-2 drugs that are novel and very specific in mechanism of action.

**Figure 1:**
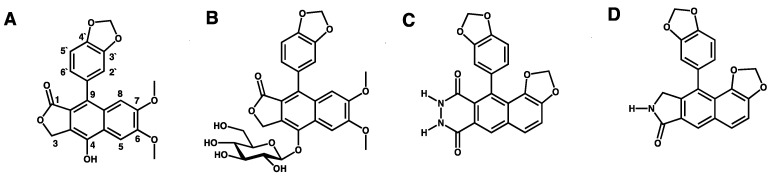
Structures of the studied compounds. (**A**) Diphyllin, (**B**) cleistanthin B, (**C**) helioxanthin 8-1, and (**D**) helioxanthin 5-4-2.

An essential step in the infection cycle of enveloped viruses is a frequently pH-dependent fusion of viral envelopes with host cell endosomal membranes. Cell entry of enveloped viruses takes place by endocytosis. During endocytosis, a vacuolar ATPase (V-ATPase) acts as a proton pump to increase the proton concentration and so cause the acidification of inner endosome compartments [6]. Therefore, inhibitors of V-ATPase have the potential to act as de facto inhibitors, as well of the pH-dependent fusion of viral envelopes with host cell endosomal membranes. Given this, the V-ATPase represents an attractive novel and specific new antiviral drug target, and small molecule inhibitors represent a promising group of potential novel and specific new antiviral agents [7]. For this reason, we chose to investigate diphyllin and its analogues. Diphyllin and the diphyllinosides are natural compounds of the arylnaphtalide lignan family, extracted from the plants *Cleistanthus collinus*, *Justicia gendarussa*, *Haplophyllum bucharicum,* plus some others, which are widely used in traditional Chinese medicine [8]. Furthermore, these natural compounds are known V-ATPase inhibitors with the ability to suppress acidification in human osteoclasts [9], to decrease V-APTase activity and infectivity in gastric adenocarcinoma cells [10], and to inhibit the replication cycles of many fungal [11], bacterial [12], and viral pathogens. Of particular relevance here, diphyllin and the diphyllinosides have already been demonstrated to exhibit some inhibitory effects in vitro and in vivo against several enveloped viruses, such as influenza virus [13], HIV-1 [14], Zika virus (ZIKV) [6], feline infectious peritonitis virus (FIPV) [7], and vesicular stomatitis virus (VSV) [15]. Apart from diphyllin and diphyllinosides, numerous other structurally related compounds have been identified with interesting biological properties [15], as exemplified by helioxanthins, whose molecular mechanisms of action are still poorly understood [16]. Here we evaluate diphyllin for its ability to inhibit replication of SARS-CoV-2 in vitro and compare its antiviral and cytotoxic properties with those of diphyllinoside cleistanthin B (diphyllin-4-β-D-glucopyranoside), helioxanthin 8-1, and helioxanthin 5-4-2, whose antiviral activities have been reported previously [17,18,19] (Figure 1).

## 2. Materials and Methods

### 2.1. Compounds, Virus, and Cell Lines

Diphyllin was synthesized by Apigenex (Prague, Czech Republic), cleistanthin B was obtained from WuXi AppTec (Tianjin, China), helioxanthin 8-1 and helioxanthin 5-4-2 were purchased from MedChemExpress (Stockholm, Sweden). All compounds were solubilized in dimethyl sulfoxide (DMSO) as 10 mM stock solutions. SARS-CoV-2 (strain SARS-CoV-2/human/Czech Republic/951/2020 was isolated from a clinical sample at the National Institute of Health, Prague, Czech Republic), and kindly provided by Dr. Jan Weber, Institute of Organic Chemistry and Biochemistry, Prague, Czech Republic. This was passaged five times through Vero E6 cells (ATCC CRL-1586) for multiplication before use. Vero cells (ATCC CCL-81, African Green Monkey, adult kidney, epithelial cells) were used for antiviral, cytotoxicity, and immunostaining assay studies. Otherwise, Vero E6 cells (ATCC CRL-1586) were used again for plaque assays. Both Vero cell lines were grown in Dulbecco’s modified Eagle’s medium (DMEM) supplemented with 10% newborn calf serum, 100 U/mL penicillin, 100 µg/mL streptomycin, and 1% glutamine (Sigma–Aldrich, Prague, Czech Republic). Colorectal adenocarcinoma cells (CaCo-2, ATCC HTB-37) were used for further characterization of the antiviral and cytotoxicity effects of diphyllin, and were grown in DMEM medium, containing, 20% newborn calf serum with 100 U/mL penicillin, 100 µg/mL streptomycin, and 1% glutamin (Sigma–Aldrich, Prague, Czech Republic).

### 2.2. Cytotoxicity Assay

To determine the cytotoxicity of diphyllin, Vero cells (ATCC CCL-81), Vero E6 cells (ATCC CRL-1586), and CaCo-2 cells (ATCC HTB-37) were seeded in 96-well microtitration plates (2 × 10^4^ cells/well) and incubated for 24 h. After incubation, diphyllin was added to the cells, in a concentration range from 0 to 100 µM, and the treated cells were further cultivated for 48 h at 37 °C. The cytotoxicities of cleistanthin B, helioxanthin 8-1 and helioxanthin 5-4-2 were tested in a similar way but only in Vero cells (ATCC CCL-81). All cytotoxicities were measured in terms of cell viability as determined with Cell Counting Kit-8 (Dojindo Molecular Technologies, Munich, Germany), used according to manufacturer’s instructions. The respective concentrations of each compound under investigation were determined that reduced cell viability by 50% (CC_50_ values).

### 2.3. Antiviral Assays

The antiviral efficacies of diphyllin, cleistanthin B, helioxanthin 8-1, and helioxanthin 5-4-2 were initially tested at a single concentration of 50 µM/well with Vero cells (ATCC CCL-81) using a viral titer reduction assay. Since Vero cells were observed to be the most resistant to diphyllin cytotoxicity, we chose this cell line to perform crucial antiviral cell-based assays. Growth medium was aspirated from Vero confluent monolayers cultivated in 96-well microtiter plates for 24 h and subsequently replaced with fresh medium (200 µL) containing the compounds under investigation and SARS-CoV-2 at a multiplicity of infection (MOI) of 0.1. Drug dosing and virus infection were performed simultaneously. Vero cells treated with either DMSO 0.5% (*v*/*v*) or remdesivir (50 µM) were used as controls. Drug-treated virus-infected cells were then cultured for 48 h at 37 °C. Viral titers were determined from collected supernatant media by a plaque assay.

In order to study the dose-response effects of diphyllin and cleistanthin B, the same experimental procedure was performed, as described above, with slight modifications. Growth medium was aspirated from Vero cell monolayers, replaced with fresh medium (200 µL) containing the compounds under evaluation in the concentration range 0 to 50 µM and SARS-CoV-2 at an MOI of 0.1, then cells were cultivated for 48 h at 37 °C. Viral titers were determined from the collected supernatant media and used to construct dose-dependent curves from which 50% effective concentrations (EC_50_ values) were calculated. The anti- SARS-CoV-2 effect of diphyllin was studied also in CaCo-2 cells (ATCC HTB-37). Owing to the higher sensitivity of CaCo-2 cells to diphyllin cytotoxity, only lower concentrations were tested (i.e., 1.5, and 3.0 µM).

For testing whether or not diphyllin retained its antiviral activity even when added at various time intervals after infection, we performed four independent post-treatment assays, each differing in the time of drug addition to infected Vero cells (ATCC CCL-81). The cells were seeded in 96-well plates (2 × 10^4^ cells/well) and incubated for 24 h to form a confluent monolayer. The cell monolayers were then infected with SARS-CoV-2 (MOI of 0.1). Medium containing diphyllin (at either 12.5, 25, or 50 µM) was added to infected cells 2, 4, 6, and 24 h post infection (h.p.i). Cells were then further incubated up to 48 h. Following incubation, the viral titers were determined from the collected supernatant media by plaque assay and the viral surface antigen expression visualized using an immunostaining assay.

The dependence of diphyllin antiviral efficacy on viral MOI was tested as follows. Vero cells (ATCC CCL-81) were seeded in 96-well plates (2 × 10^4^ cells per well) and incubated for 24 h to form a confluent monolayer. The obtained Vero cell monolayers were subsequently treated with a fresh medium containing diphyllin, at concentrations of 25 and 50 µM, and infected with SARS-CoV-2 at three different MOIs of 0.1, 1, and 10. The infected cells were then further incubated for 48 h. Thereafter, viral titers were determined from collected supernatants by plaque assay.

### 2.4. Plaque Assay

Plaque assays were performed in Vero E6 cells (ATCC CRL-1586), using a modified protocol originally developed by De Madrid and Porterfield [20]. In Vero E6 cells, SARS-CoV-2 forms large, circular plaques, which are regular in shape and size, and, therefore, this cell line is particularly suitable for plaque assays and subsequent viral titer quantification. Briefly, 10-fold dilutions of virus were prepared in 24-well tissue culture plates, and the cells were added to each well (0.6–1.5 × 10^5^ cells/well). After 4 h incubation, the suspension was overlaid with 1.5% (*w*/*v*) carboxymethylcellulose in DMEM. Following a 5-day incubation at 37 °C, the infected plates were washed with phosphate-buffered saline, and the cell monolayers were stained with naphthalene black. The virus titer was expressed as plaque forming units (PFU)/mL.

### 2.5. Immunofluorescence Staining

To measure the compound-induced inhibition of viral surface antigen expression, a cell-based immunostaining assay was performed as previously described [21], with minor modifications. Briefly, Vero cells (ATCC CCL-81) were seeded onto 96-well microtitration plates and 24 h later the confluent monolayers were infected with SARS-CoV-2 at MOI of 0.1. Then, diphyllin or cleistantin B were added at different concentrations to cell monolayers that were then cultured for 48 h at 37 °C. After cold acetone–methanol (1:1, *v*/*v*) fixation and blocking with 10% fetal bovine serum, cells were incubated with a rabbit (2019-nCoV) spike S1 antibody (1:50; Sino Biological, Duesseldorfer, Germany) and subsequently incubated for 1 h at 37 °C with anti-rabbit goat secondary antibodies conjugated with fluorescein isothiocyanate (FITC; 1:250; Sigma–Aldrich, Prague, Czech Republic). Cells were counterstained with 4′,6-diamidino-2-phenylindole (DAPI) (1 μg/mL) for the visualization of cell nuclei and fluorescence signals were recorded with an Olympus IX71 epifluorescence microscope.

### 2.6. Statisctics Analysis

Data were expressed as mean ± standard deviation (SD), and the significance between groups was evaluated by the unpaired parametric two-tailed t-test using GraphPad Prism 7.04 (GraphPad Software, Inc., San Diego, CA, USA). *P* < 0.05 was considered significant. EC_50_ and CC_50_ values were calculated as inflection points in sigmoidal inhibitory and cell viability curves, which were obtained by a nonlinear fit of transformed inhibitor concentrations versus normalized response using GraphPad Prism 7.04.

## 3. Results and Discussion

**Figure 2:**
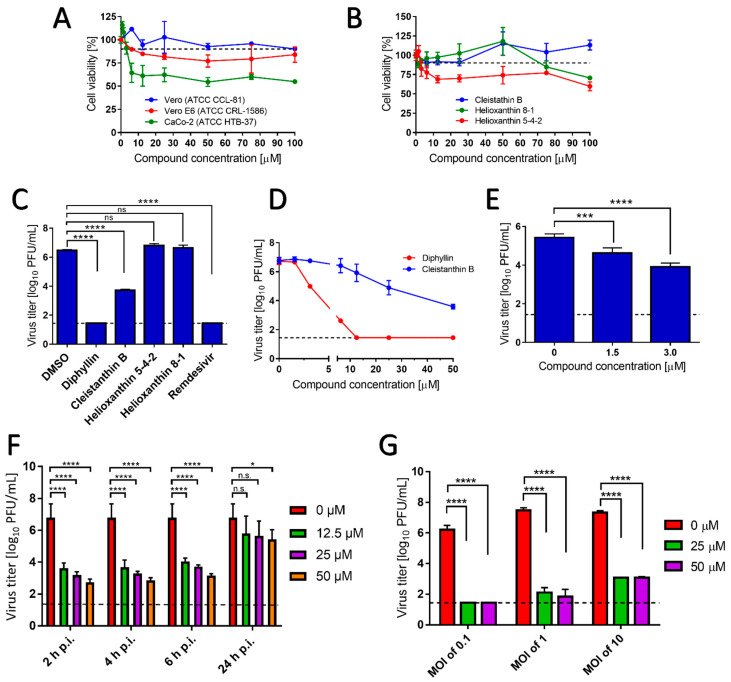
(**A**) Cytotoxicities of diphyllin in Vero, Vero E6, and CaCo-2 cells are shown and expressed as percentage of cell viability. The cells were treated with diphyllin at the indicated concentrations and incubated for 48 h. (**B**) Cytotoxicities of cleistanthin B, helixanthin 8-1, and helioxanthin 5-4-2 in Vero cells are shown. The cells were treated with the compounds at the indicated concentrations and incubated for 48 h. (**C**) Anti-SARS-CoV-2 activities of diphyllin, cleistanthin B, helixanthin 8-1, and helioxanthin 5-4-2 are shown as measured in Vero cells infected with SARS-CoV-2 (multiplicity of infection (MOI) of 0.1) and simultaneously treated with each compound under investigation (50 µM). Infected cells were incubated separately with each compound under investigation for 48 h post infection (h.p.i.) and viral titers were determined using the plaque assay. Remdesivir was used as a positive control. (**D**) Growth curves are shown of SARS-CoV-2 in Vero cells treated with diphyllin and cleistanthin B (at the indicated concentrations). Vero cells were infected with the virus (MOI of 0.1) and simultaneously treated with the compounds under investigation, then incubated for 48 h.p.i. Viral titers were quantified using the plaque assay. (**E**) Anti-SARS-CoV-2 activities are shown in CaCo-2 cells using diphyllin at the indicated concentrations. (**F**) Anti-SARS-CoV-2 activities are shown in SARS-CoV-2-infected Vero cells that were treated with diphyllin 2, 4, 6, and 24 h.p.i. (at the indicated concentrations) then incubated up to 48 h. (**G**) Anti-SARS-CoV-2 activities are shown in SARS-CoV-2-infected Vero cells (MOIs of 0.1, 1, and 10) that were treated with diphyllin (at the indicated concentrations) and then incubated for 48 h.p.i. Viral titers were determined by a plaque assay. The mean titers from three biological replicates are shown, and error bars indicate standard errors of the mean. The horizontal dashed line indicates the minimum detectable threshold of 1.44 log_10_ plaque forming units (PFU)/mL. n.s., not significant; *, *p* < 0.05; ***, *p* < 0.001; ****, *p* < 0.0001.

Diphyllin, diphyllinoside cleisthantin B, and two structurally related helioxanthins (Figure 1) were evaluated for their in vitro cytotoxic profiles, and their anti-SARS-CoV-2 activities. Diphyllin cytotoxicity was assessed in Vero, Vero E6, and CaCo-2 cells over the concentration range 0 to 100 µM at 48 h post treatment. In Vero cells, diphyllin was found well tolerated, showing a CC_50_ value of >100 µM (Figure 2A and Table 1). Diphyllin cytotoxicity in the Vero E6 cell line was greater such that cell viability declined to approx. 75% with 50 µM diphyllin although a CC_50_ value of >100 µM was still determined (Figure 2A). Diphyllin exhibited its highest cytotoxicity in CaCo-2 cells (CC_50_ 54.6 ± 4.8 µM) (Figure 2A). The cytotoxicities of cleistanthin B, helioxanthin 8-1, and helioxanthin 5-4-2 were determined only in Vero cells. Whereas cleistanthin B showed a decent cytotoxicity profile over the tested concentration range, helioxanthin 8-1 caused a decline of cell viability to 70.6 ± 2.4% at 100 µM. Treatment with helioxanthin 5-4-2 resulted in a larger decrease in cell viability to 60 ± 5.8% at 100 µM (Figure 2B).

Interestingly, Vero cells do appear to be more resistant to the cytotoxic effects of diphyllin and other diphyllin-related compounds compared to other cell types, as reported by other authors. Chen et al. [13] measured diphyllin cytotoxicity in Mardin–Darby canine kidney (MDCK) and A549 cells resulting in CC_50_ values of 3.48 and 24.01 µM, respectively. Similarly, diphyllin was shown to be cytotoxic for fcwf-4 cells (CC_50_ 5.99 µM). Notably, diphyllin-poly(ethylene glycol)-block-poly(lactide-co-glycolide) (PLGA) nanoparticles were much less cytotoxic (CC_50_ of 77.3 µM) in comparison to free diphyllin [7]. On the other hand, Asano et al. [15] reported that minimum cytotoxic concentrations of diphyllin, diphyllin apioside, diphyllin apioside-acetate, and justicidins A, C, and D (Figure 1 and Figure S1) were 63 µg/mL in RL-33 cells. Approximately 2-4-fold lower toxicities were observed using justicidinoside A, B, and C (Figure S1) with minimum cytotoxic concentrations of 125 and 250 µg/mL identified in RL-33 cells. The cytotoxicity of helioxanthin 8-1 was studied in dstet5 cells and CC_50_ values were determined from 18 µM to 45 µM [19]. The CC_50_ values of helioxanthin 5-4-2 in HepG2 cells reached single-digit micromolar values [18].

In evaluating the antiviral potency of diphyllin, cleistanthin B, helioxanthin 8-1, and helioxanthin 5-4-2, Vero cells infected with SARS-CoV-2 (MOI of 0.1) were treated with a single concentration of each compound under investigation (50 µM), then cells were cultivated for 48 h.p.i. Under these conditions, diphyllin completely inhibited virus replication, resulting in a viral titer decrease of 10^6^-fold compared with the situation with virus-infected mock-treated cells. This effect was comparable with the antiviral effect of remdesivir used as a positive control (Figure 2C). The antiviral effect of cleistanthin B was somewhat lower than that of diphyllin resulting in a decrease in viral titer of 10^3^-fold. Interestingly, helioxanthin 8-1 and helioxanthin 5-4-2 appeared to be inactive against SARS-CoV-2 at the tested concentration and were therefore excluded from further antiviral analyses (Figure 2C). This was somewhat surprising to us given that the tested helioxanthin analogues have been described elsewhere to exhibit broad-range antiviral activities [16,18,19].

**Figure 3:**
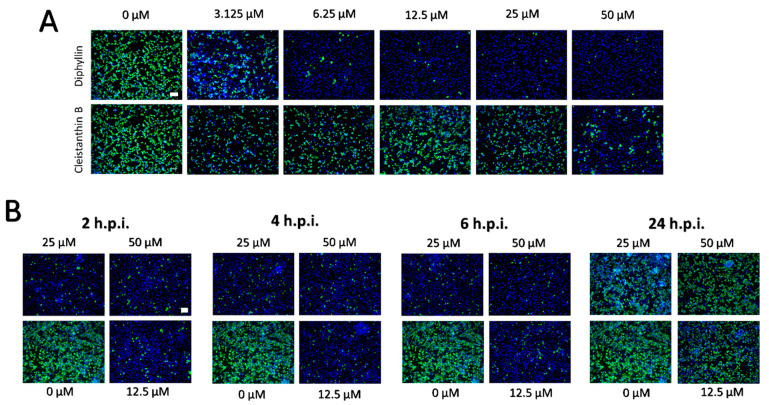
(**A**) Inhibition of SARS-CoV-2 viral antigen expression by diphyllin and cleistanthin B. Compound-treated and virus-infected Vero cells cultivated for 48 h.p.i. were fixed on slides, then stained with a SARS-CoV-2 specific antibody labeled with FITC (green), and counterstained with DAPI (blue). (**B**) Immunofluorescence staining of Vero cell cultures infected with SARS-CoV-2 and treated with diphyllin at 2, 4, 6, and 24 h.p.i. The immunofluorescent staining procedure was identical as in (**A**). Scale bars; 50 µM.

The dose-dependent anti- SARS-CoV-2 effects of diphyllin and cleistanthin B (0 to 50 µM) were quantified in Vero cells by plaque assay 48 h.p.i. Using this assay, diphyllin was observed to reduce viral titers with an EC_50_ value of 1.92 µM (Figure 2D, Table 1). Diphyllin notably mediated complete inhibition of viral replication at ≥12.5 µM. In comparison, cleistanthin B was observed to reduce viral titers with an EC_50_ value of 6.51 µM indicating that this compound is 3.4-fold less potent than diphyllin (Figure 2D, Table 1). In addition, cleistanthin B was only able to reduce viral titer by 10^3^-fold at the highest tested concentration of 50 µM 48 h.p.i. (Figure 2D). These dose-dependent antiviral effects of diphyllin and cleisthantin B were further confirmed by immunofluorescence staining used to monitor the level of coronavirus spike S1 antigen expression in Vero cells as a measure of both viral infectivity and successful viral replication in vitro (Figure 3A). Although S1 protein was highly expressed in virus-infected mock-treated cells, S1 protein fluorescence was observed to decrease towards virtually undetectable in Vero cell monolayers 48 h.p.i. when infected cells were treated with ascending concentrations of diphyllin (12.5, 25, and 50 µM). In virus-infected cells treated with cleistanthin B, the decrease in S1 antigen expression was less obvious compared with diphyllin (Figure 3A), in agreement with results from virus titer reduction assays (Figure 2D).

Owing to the superior antiviral activity of diphyllin, further anti- SARS-CoV-2 studies were performed only with this compound. Initially, the anti- SARS-CoV-2 effect of diphyllin was assessed in CaCo-2 cells, which represents another highly susceptible cell line frequently used for SARS-CoV-2 multiplication, virus pathogenesis studies, and antiviral assays. Given that this cell line appeared considerably more sensitive to diphyllin cytotoxicity than Vero cells, antiviral studies were only performed using lower diphyllin concentrations (1.5 and 3.0 µM) resulting in only a moderate but nonetheless statistically significant decrease in viral titer (approximately 1 and 2 orders of magnitude, respectively) compared the situation with control cells (Figure 2E). Following this, time-of-addition assays were performed in which diphyllin was administered in ascending concentrations (12.5, 25, and 50 µM) between 2, 4, 6, and 24 h.p.i. Even up to 6 h.p.i., viral titers were decreased up to 4 orders of magnitude. Moreover, a statistically significant decline in viral titers was even observed in virus-infected Vero cells, even when diphyllin was added at a concentration of 50 µM 24 h.p.i. (Figure 2F). The anti-SARS-CoV-2 time-of-addition effects of diphyllin were further confirmed by means of viral antigen immunostaining (Figure 3B). Finally, the anti-SARS-CoV-2 effect of diphyllin (at 25 and 50 µM) was further tested using viral MOIs of 1 and 10, instead of the MOI of 0.1 used previously here (see above). Although, the antiviral activity of diphyllin at high MOIs was weaker than with a MOI of 0.1, nevertheless the compound reduced viral titers more than 10^6^-fold (MOI of 1) and 10^5^-fold (MOI of 10) 48 h.p.i (Figure 2G). These data clearly demonstrate the strong antiviral potency of diphyllin given that diphyllin is able to suppress SARS-CoV-2 replication even in a single-cycle infection model at high MOI.

Our data here strongly interlock to support the view that diphyllin is a potent agent against anti-SARS-CoV2. Elsewhere, diphyllin has been described to inhibit endosomal acidification in fcwf-4 cells, resulting in nanomolar suppression of the replication of FIPV, another member of the *Coronaviridae* family [7]. Diphyllin has also been shown to be potent in vitro against influenza virus in MDCK cells [13] and against HIV-1 in human peripheral blood mononuclear cells (PBMC) [17]. Furthermore, a combination treatment of diphyllin with oseltamivir and amantadine has been shown to have more anti-influenza efficacy in cell cultures than diphyllin itself [13]. In comparison, diphyllin derivatives, justicidin A and B, differing from diphyllin by a C4-methoxy group or C4-hydrogen (in place of the C4-hydroxy group) (Figure 1 and Figure S1), have been shown, respectively, to exhibit anti-VSV effects when tested in RL-33 cells. Although, justicidins C and D with a C3-carbonyl lactone ring (instead of the C1-carbonyl lactone ring in diphyllin) (Figure 1 and Figure S1) were found to be approximately 100-fold less active [15].

Turning to diphyllinosides, these diphyllin derivatives differ from diphyllin in the conjugation of pentofuranose or hexopyranose rings of different types or positions and have also been found to possess potent antiviral activities [6,14,15,17]. For instance, diphyllin apioside and diphyllin apioside-acetate, with pentofuranose rings attached to C4 (Figure S1), have been found equivalent in anti-VSV assays compared with free diphyllin [15]. In contrast, justicidinosides A–C, characterized by a β-glucopyranose (Glc) on C6’ of the 1,3-benzodioxole moiety (Figure S1), have been found 100–1000-fold less potent than diphyllin although with better cytotoxicity profiles [15]. Otherwise, patentiflorin A (Figure S1) has been shown to block ZIKV replication in both human and monkey cell lines and exhibits broad-spectrum antiviral activities in vitro across the *Flaviviridae* family, even preventing mortality in a rodent in vivo model of ZIKV infection [6]. This same patentiflorin A also displayed nanomolar activity against four clinical HIV-1 isolates, using a standardized human PBMC assay, which was 20–30 times more potent than either free diphyllin or cleistanthin B [17]. Finally, justiprocumin B (Figure S1) was also found to exhibit broad spectrum nanomolar inhibition of HIV strains in PBMC, including of those strains resistant to reverse transcriptase inhibitors zidovudine and nevaripine [14]. Nevertheless, what appears to be the case for these diphyllinosides is that the active component is diphyllin while the sugar moieties act to modulate (decrease or increase) the antiviral potency [6,15].

## 4. Conclusions

The presented study demonstrates that both diphyllin and cleistanthin B possess low-micromolar anti- SARS-CoV-2 activities in Vero cells with negligible cytotoxicities in both cases. The anti- SARS-CoV-2 effect of diphyllin was approximately 4-fold stronger than that of cleistanthin B and caused a statistically significant suppression of viral replication, even if applied at various times post-infection and at high MOIs. Diphyllin also showed an antiviral effect in CaCo-2 cells. Our results indicate, that diphyllin itself, and to a lesser extent cleistanthin B, are potentially interesting natural compounds with strong antiviral potency against SARS-CoV-2. However, both compounds suffer from poor solubility and correspondingly poor in vivo bioavailability. These problems will need to be addressed if either or both compounds are to be developed for clinical use.

## Figures and Tables

**Table 1:** Antiviral and cytotoxicity characteristics of diphyllin and cleistanthin B in Vero cells.

Compound	EC_50_ (µM) ^1,2^	CI ^3^	CC_50_ (µM) ^1^	SI ^4^
diphyllin	1.92	1.52 to 2.43	>100	>52.08
cleistanthin B	6.51	4.71 to 8.98	>100	>15.36
helioxanthin 8-1	>50	N. D.	>100	N. D.
helioxanthin 5-4-2	>50	N. D.	>100	N. D.

^1^ Determined from three independent experiments. ^2^ Defined as the critical concentration at which a 50% reduction in viral titers is observed as calculated by the inflection point of sigmoidal dose-response curves, generated using GraphPad Prism 7.04 (GraphPad Software, Inc., USA) by nonlinear fitting of transformed inhibitor concentrations versus normalized response. ^3^ confidence interval (CI) calculated using GraphPad Prism 7.04 (GraphPad Software, Inc., USA). ^4^ selectivity index (SI) = CC_50_/EC_50_. N. D.; not determined.

## Data Availability

The datasets generated and analyzed during the current study are available from the corresponding author on reasonable request.

## Supplementary Materials

The following are available online at https://www.mdpi.com/2076-2607/9/3/471/s1, Figure S1: Structures of diphyllin analogues and diphyllinosides.
