# Supplementary material for: Antiviral Activity of Vacuolar ATPase Blocker Diphyllin against SARS-CoV-2
Source: Microorganisms. 2021 Feb 25;9(3):471. doi: 10.3390/microorganisms9030471 (PMC7996309; doi:10.3390/microorganisms9030471)
Supplement: Supplementary file 1 [file microorganisms-09-00471-s001.pdf]

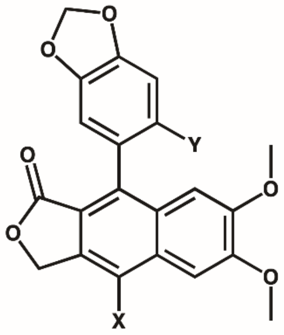

justicidin A; X = OMe, Y = H
justicidin B; X = H, Y = H

justicidinoside B; X = OMe, Y = Glc
justicidinoside C; X = H, Y = Glc

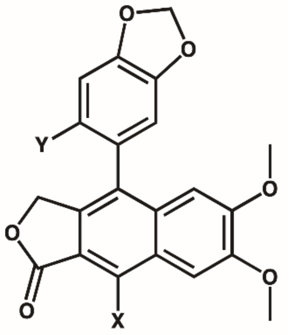

justicidin C; X = OMe, Y = H

justicidinoside A; X = OMe, Y = Glc

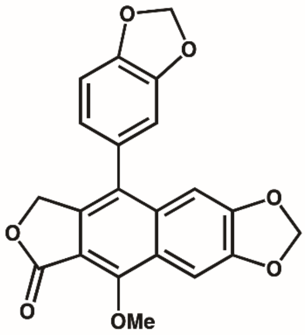

justicidin D

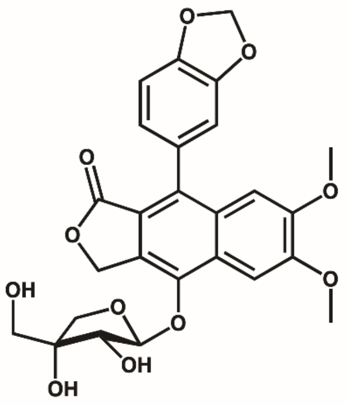

diphyllin apioside

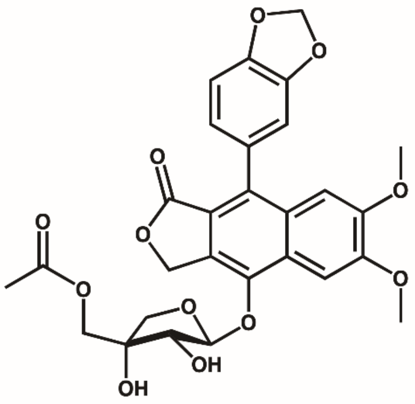

diphyllin apioside-acetate

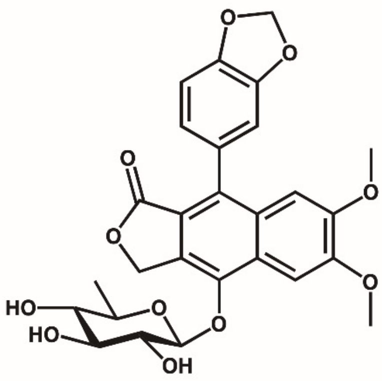

patentiflorin A

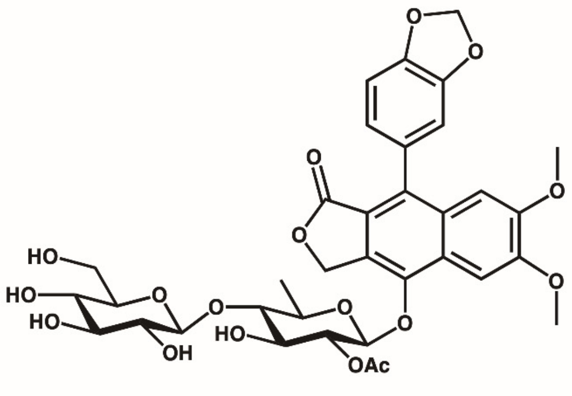

justiprocumin B

Figure S1. Structures of diphyllin analogues and diphyllinosides.
